# Cutaneous Granulomatosis Revealing Whipple’s Disease: Value of *Tropheryma whipplei* Polymerase Chain Reaction Assay for the Diagnosis

**DOI:** 10.3390/pathogens10111438

**Published:** 2021-11-05

**Authors:** Souheil Zayet, Pierre Isnard, Jacinta Bustamante, David Boutboul, Sarra Abroug, Nabil Belfeki

**Affiliations:** 1 Department of Infectious Diseases, Nord Franche-Comté Hospital, 90400 Trévenans, France; 2 Department of Pathology, Necker Hospital for Sick Children, Assistance Publique des Hopitaux de Paris (Ap-HP), 75015 Paris, France; pierre.isnard@aphp.fr; 3 Department of Cellular Biology, University of Paris, 75105 Paris, France; jacinta.bustamante@inserm.fr; 4 Laboratory of Human Genetics of Infectious Disease Laboratory, Necker Branch, INSERM U1163, Necker Hospital for Sick Children, 75015 Paris, France; 5 St. Giles Laboratory of Human Genetics of Infectious Diseases, Rockefeller Branch, The Rockefeller University, New York, NY 10065, USA; 6 Study Center for Primary Immunodeficiencies, Necker Children Hospital, Assistance Publique des Hopitaux de Paris (AP-HP), 75015 Paris, France; 7 Department of Clinical Immunology, Saint Louis Hospital, Assistance Publique des Hôpitaux de Paris, University of Paris, 75010 Paris, France; david.boutboul@aphp.fr; 8 Department of Internal Medicine, Groupe Hospitalier Sud Ile de France, 77000 Melun, France; sarra.abroug@ghsif.fr

**Keywords:** granulomatosis, polymerase chain reaction, skin, Whipple’s disease, *Tropheryma whipplei*

## Abstract

Whipple’s Disease is a rare systemic infectious disease caused by the ubiquitous actinomycetes *Tropheryma whipplei (T. whipplei).* We report herein a rare case of a cutaneous granulo matosis with hypercalcemia as an unusual presenting feature of Whipple’s disease. The diagnosis of the bacteria was obtained from skin and inguinal lymph node biopsy (16 rDNA PCR screening and histological examination using PAS staining). *T. whipplei* was also identified on saliva and stool specimens, using specific PCR and colonic biopsies. Treatment with hydroxychloroquine and doxycycline allowed a rapid resolution of symptoms with a complete recovery.

## 1. Introduction

Whipple’s disease (WD) is a rare chronic systemic infection caused by the slow-growing and Gram-positive bacterium *Tropheryma whipplei (T. whipplei). T. whipplei* is a pathogen responsible for a wide range of clinical manifestations in humans, beginning with acute infections, chronic isolated infections, and classic WD [1,2,3]. Asymptomatic carriage has been also reported. The classic form usually involves the intestinal tract, with diarrhea and weight loss as the main clinical features, associated with recurrent arthritis, mostly polyarthritis. However, extra intestinal manifestations can also include extra intestinal organs, such as endocarditis uveitis, neurologic, and dermatologic manifestations, which is exceptional [2,4]. In some rare cases, it can also cause cutaneous lesions, such as subcutaneous nodules, erythema nodosum or vasculitis [5]. The genetic background has long been suggested by the strong male predominance and the reported association with the antigens HLA B27, DRB1*13 or DQB1*06. In 2018, a single rare monoallelic missense mutation was identified in interferon regulatory factor 4 (IRF4), a transcription factor involved in immunity, which showed that autosomal dominant (AD) IRF4 deficiency can underlie WD by haploinsufficiency with incomplete penetrance [6]. 

We report herein a case of WD with cutaneous granulomatosis and bilateral lymphadenopathy in a patient presenting with chronic diarrhea. The WD diagnosis was raised due to the presence of suggestive histological features and confirmed by polymerase chain reaction (PCR) in multiple sites (biopsy lymph nodes completed with saliva and stool).

## 2. Observation

**Figure 1:**
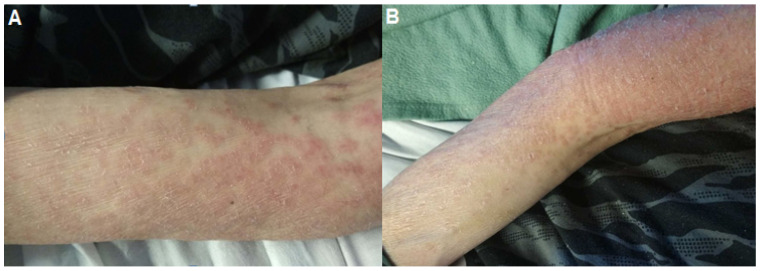
The lesions developed as erythematous scaly follicular papules (**A**) and plaques over both forearms (**B**).

**Figure 2:**
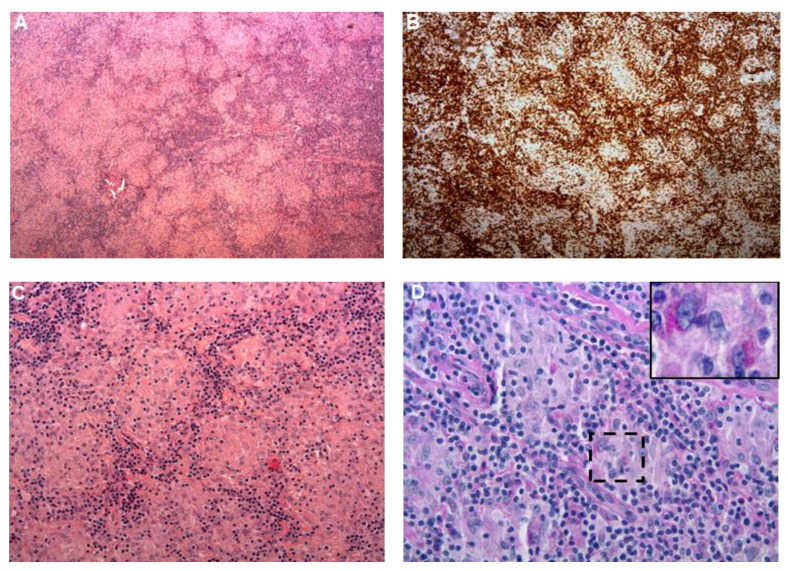
(**A**). Light microscopy using HES staining (×50) showed a complete effacement of nodal architecture with granulomatous inflammation characterized by the formation of multiple granulomas of various size. (**B**) Immunochemistry (×50) using anti-CD5 antibody showed an important T-cell infiltrate surrounding granulomas. (**C**) Light microscopy using HES staining (×200) showed granulomas composed of aggregates of epithelioid histiocytes, with a peripheral cuff of lymphocytes. (**D**) Light microscopy using PAS staining revealed PAS positive cytoplasmic granulation within histiocyte suggestive of Whipple’s disease (black square).

A 54-year-old Caucasian patient from a non-consanguineous family with a past history of arterial hypertension arrived at the emergency room presenting with fatigue, weight loss, and gastro-intestinal (GI) symptoms including nausea and vomiting, abdominal pain and intermittent fluid diarrhea that had been occurring for two years. He did not report any recent travel. He did not smoke tobacco, drink alcohol, nor use illicit drugs. At admission, a physical examination was performed that revealed a blood pressure of 132/100 mmHg, a regular heartbeat (112 beats/min), afebrile (36.7 °C) with a body mass index (BMI) of 18 kg/m^2^, and erythematous scaly plaques in upper limbs (Figure 1). Abdominal examination revealed hepatosplenomegaly with bilateral (firm, elastic, mobile, and painless) inguinal lymph nodes, without fistula or abscess. Abdominal computed tomography (CT) imaging showed multiple inguinal and intra-abdominal enlarged hypodense lymph nodes, with hepathomegaly (17 cm). Respiratory and neurologic examinations were normal. Laboratory findings showed a mild normocytic normochromic anemia of 11 g/dl (normal range 12–16 g/dl), normal white cell count of 6.2 Giga /L (normal range 4–10 Giga /L), normal count of neutrophils (5100/mm^3^, normal range 2500–7000/mm^3^) and monocytes (260/mm^3^, normal range 100–700/mm^3^) with lymphopenia of 840/mm^3^ (normal range 1500–4000/mm^3^). Platelets were at 282 Giga /L (normal range 150–450 Giga /L). C-reactive protein was high at 73 mg/L (normal range < 5 mg/L), and liver function was normal. Serum electrolytes were consistent with expected hypercalcemia at 4.15 mmol/L (normal range 2.25–2.62 mmol/L). An acute kidney failure with creatinine at 207 µmol/L (normal range 60–120 mmol/L using the CKD-EPI) was noted. PTH, PTH-rp and 25–OH-vitamine D levels were normal. Serum protein electrophoresis showed hypoprotidemia at 48.6 g/L (normal range 60–80 g/L) with hypoalbuminemia at 25.6 (normal range 36–50 g/L) associated with hypogammaglobulinemia at 6.9 g/L (normal range 8–16 g/L). Lymphocytes immunophenotyping did not show any B or T clonal circulating lymphoma and the percentage of T, B and NK was normal. Subsets of IgG (at 5.70 g/L), IgA (at 1.59 g/L), IgM (0.23 g/L) and IgE were in the range of the matched age. Immunoelectrophoresis did not show monoclonal gammopathy, medullary smear and bone marrow biopsy were normal, which ruled out lymphoma. Supportive treatment began (simultaneous intravenous (IV) administration of fluids associated with biphosphonates and steroids). Hepatitis B and C, HIV and syphilis serologies were negative. The QuantiFERON-TB Gold test was negative. Direct examination and culture of the stool sample were negative. Skin and a right lymph node biopsy were performed. Upon histological examination, light microscopy showed granulomas composed of aggregates of epithelioid histiocytes, with no necrosis and negative Ziehl–Neelsen staining (Figure 2). Microscopic examination of sputum for acid-fast bacilli (AFB) was negative. Biopsy results were also negative for *Mycobacterium tuberculosis* in culture and rt-PCR. Finally, 16 rDNA PCR testing of the inguinal lymph node biopsy revealed *T. whipplei,* which was confirmed in a positive-specific PCR performed on saliva and stool samples. A classic WD secondary to *T. whipplei* was diagnosed from the results of microbiological testing (PCR) and the histological examination using periodic acid-Schiff (PAS) staining (Figure 2). Colonoscopy was macroscopically normal and colonic biopsies showed negative PAS stains, but PCR *T. whipplei* was positive. Upper endoscopy with duodenal biopsies and *T. whipplei* PCR were negative. 

Brain magnetic resonance imaging (MRI) and lumbar puncture were normal. Cardiac MRI was normal. Whole exome-sequencing was performed, but no mutation in IRF4 was identified. The patient started hydroxychloroquine (400 mg/day) and doxycycline (200 mg/day). The outcome was favorable, with a progressive improvement of his general status. Diarrhea was discontinued, CT control showed reduction of lymph node volume as well as liver enlargement. Furthermore, calcium level was also normalized. The patient was discharged on the same oral treatment for 12 months with a complete recovery.

## 3. Discussion

We report herein a new patient with typical WD symptoms of diarrhea, weight loss, granulomatous lymphadenopathy and cutaneous features. The cutaneous lesions in WD are infrequent and unspecific [7,8], except the skin hyperpigmentation and symmetrical panniculitis [9]. In this case, a biopsy revealed a florid septal panniculitis with infiltration of the septa by foamy macrophages containing intracellular granules that were strongly stained with PAS reagent [9]. Cutaneous granulomatosis related to WD is also a rare clinical feature previously reported in some cases [10,11]. Erythema nodosum-like lesions can be described in WD treated with antibiotics as features of immune reconstitution inflammatory syndrome [12,13]. 

In our case, a lymph node biopsy showed granulomatous lymphadenopathy without necrosis. Several etiologies such as tuberculosis, HIV, sarcoidosis and lymphoma were initially suspected [14]. Ziehl–Neelsen staining can discriminate between intracellular *T. whipplei* and *Mycobacterium* spp., as both are positive with PAS staining [4], but only mycobacteria are positive with Ziehl–Neelsen staining. Immunophenotyping and bone marrow biopsy ruled out lymphoma. Only PCR testing on lymph nodes biopsies revealed *T. whipplei* DNA and confirmed the diagnosis and thus ruled out systemic sarcoidosis. This case illustrates a granulomatous dermal reaction related to classic WD. In a similar case of a patient with isolated peripheral lymphadenopathy in absence of GI symptoms, Walters et al. were able to diagnose Whipple’s early in the course of clinical disease from specific PCR in lymph node biopsy [15]. The gold standard for WD diagnosis used to be PAS staining of duodenal biopsy specimens, but PAS staining has a poor specificity and sensitivity [4]. Thus, 16S rDNA PCR may be a useful diagnostic tool for the identification of the causative organism by detecting and identifying bacterial nucleic acid in specimens, especially in *T. Whipplei* infection [16]. Therefore, for patients with clinically suspected WD and normal duodenal results, sampling of extra-intestinal tissue and fluids for PAS staining and *T. whipplei*-specific PCR is required and may be essential for the diagnosis. In a large retrospective study including more than 27,000 samples, Edouard et al. [17] observed that among all the positive samples, stool and skin biopsy samples exhibited a higher prevalence of positivity by rt-quantitative PCR (qPCR), at 10.07% and 15.4%, respectively. In a second cohort of 191 patients with confirmed classic WD, Gunther et al. [18] concluded that this disease can be diagnosed with PAS stains in more than 90% of patients. The remaining cases were detected via PCR of duodenal specimens and/or extra-intestinal fluids, as was performed on our patient. In the same study, the diagnosis was established in only one case, by lymph node biopsy and skin biopsy, respectively. In the same study, less than 5% of patients did not present any a positive test from the duodenum, and the WD diagnosis was set based on extra-intestinal tissues (with two patients on colonic biopsies), as for our case. Of these nine patients with a negative PAS and PCR from the duodenum, three had previously received antimicrobial drugs and four displayed no GI symptoms. In both patients with initially normal duodenal findings and solely positive *T. whipplei*-specific PCR results of extra-duodenal specimens, repeated duodenal biopsies showed WD-like histological changes, and positive *T. whipplei*-specific PCR results were obtained. In these patients, *T. whipplei*-specific immunohistochemistry could be an excellent diagnostic test with very high specificity and sensitivity which allows for the identification of *T. whipplei* in PAS and PCR-negative specimens [19,20]; otherwise, repeated duodenal biopsies with PAS and PCR detection are also required [18,21].

## 4. Conclusions

Cutaneous granulomatosis related to WD is rare. The WD assessment should be considered in the case of patients with impaired general conditions, chronic diarrhea and lymph node enlargement. Given the inadequate sensitivity of PAS stain and in order to contribute to earlier diagnosis, we suggest specific PCR for *T. whipplei* on lymph nodes biopsies. 

## Data Availability

Data available on request due to privacy restrictions. The data presented in this case study are available on request from the corresponding author.

## References

[1] Antunes C., Singhal M. (2021). Whipple Disease. StatPearls.

[2] Marth T., Moos V., Müller C., Biagi F., Schneider T. (2016). Tropheryma whipplei infection and Whipple’s disease. Lancet Infect. Dis..

[3] Boumaza A.F., Arrindell J., Ben Azzouz E., Desnues B. (2021). Phenotypic diversity of Tropheryma whipplei clinical isolates. Microb. Pathog..

[4] Dolmans R.A.V., Boel C.H.E., Lacle M.M., Kusters J.G. (2017). Clinical Manifestations, Treatment, and Diagnosis of Tropheryma whipplei Infections. Clin. Microbiol. Rev..

[5] Krusche M., Boro D., Bertolini J., Kötter I. (2019). Rare erosive arthritis and dermatitis syndrome in Whipple’s disease. Z. Rheumatol..

[6] Guérin A., Kerner G., Marr N., Markle J.G., Fenollar F., Wong N., Boughorbel S., Avery D.T., Ma C.S., Bougarn S. (2018). IRF4 haploinsufficiency in a family with Whipple’s disease. eLife.

[7] Borretta L., Walsh N.M., Bakowsky V., Arnason T., Croul S., Pasternak S. (2021). A Case of Whipple Disease With Cutaneous Manifestations. Am. J. Dermatopathol..

[8] Canal L., de la Fuente D., Rodriguez-Moreno J., Penin R.M., Marcoval J. (2014). Specific cutaneous involvement in Whipple disease. Am. J. Dermatopathol..

[9] Friedmann A.C., Perera G.K., Jayaprakasam A., Forgacs I., Salisbury J.R., Creamer D. (2004). Whipple’s disease presenting with symmetrical panniculitis. Br. J. Dermatol..

[10] Frenk E., Merot Y., Perez I., Chamot A.M., Gerster J.C. (1991). Whipple’s disease with sarcoidosis-like cutaneous manifestations. Ann. Dermatol. Venereol..

[11] Cho C., Linscheer W.G., Hirschkorn M.A., Ashutosh K. (1984). Sarcoidlike granulomas as an early manifestation of Whipple’s disease. Gastroenterology.

[12] Sanchez A., Del Giudice P., Mantion C., Mazellier S., Boukari F., Roger P.-M., Courjon J. (2021). Erythematous skin nodules during treatment of Whipple’s disease. Infect. Dis. Now.

[13] Schaller J., Carlson J.A. (2009). Erythema nodosum-like lesions in treated Whipple’s disease: Signs of immune reconstitution inflammatory syndrome. J. Am. Acad. Dermatol..

[14] Totschnig D., Seitz T., Zoufaly A., Hagenauer-Drektraan S., Wenisch C. (2021). Whipple’s disease diagnosed in a patient with suspected sarcoidosis. Int. J. Infect. Dis. IJID Off. Publ. Int. Soc. Infect. Dis..

[15] Walters S., Valliani T., Przemioslo R., Rooney N. (2014). Whipple’s disease: An unexpected finding in a peripheral lymph node biopsy. Lancet Lond. Engl..

[16] Geissdörfer W., Moos V., Moter A., Loddenkemper C., Jansen A., Tandler R., Morguet A.J., Fenollar F., Raoult D., Bogdan C. (2012). High frequency of Tropheryma whipplei in culture-negative endocarditis. J. Clin. Microbiol..

[17] Edouard S., Fenollar F., Raoult D. (2012). The rise of Tropheryma whipplei: A 12-year retrospective study of PCR diagnoses in our reference center. J. Clin. Microbiol..

[18] Günther U., Moos V., Offenmüller G., Oelkers G., Heise W., Moter A., Loddenkemper C., Schneider T. (2015). Gastrointestinal diagnosis of classical Whipple disease: Clinical, endoscopic, and histopathologic features in 191 patients. Medicine.

[19] Lepidi H., Fenollar F., Gerolami R., Mege J.-L., Bonzi M.-F., Chappuis M., Sahel J., Raoult D. (2003). Whipple’s disease: Immunospecific and quantitative immunohistochemical study of intestinal biopsy specimens. Hum. Pathol..

[20] Baisden B.L., Lepidi H., Raoult D., Argani P., Yardley J.H., Dumler J.S. (2002). Diagnosis of Wihipple disease by immunohistochemical analysis: A sensitive and specific method for the detection of Tropheryma whipplei (the Whipple bacillus) in paraffin-embedded tissue. Am. J. Clin. Pathol..

[21] Kutlu O., Şengiz E.S., Gökden Y., Kandemir Ö., Tükek T. (2020). Whipple′s Disease: A Case Report. Med. Princ. Pract..

